# Prevalence of Proteinuria in Dogs With Immune‐Mediated Disease

**DOI:** 10.1111/jvim.70162

**Published:** 2025-06-08

**Authors:** James C. Barton, Alexander J. German, Erin M. O'Connell

**Affiliations:** ^1^ Small Animal Teaching Hospital The University of Liverpool Liverpool UK

**Keywords:** autoimmunity, glomerulopathy, glucocorticoids, pyrexia, urine protein–creatinine ratio (UPCR)

## Abstract

**Background:**

Proteinuria is associated with autoimmune diseases in humans. There is minimal evidence in the veterinary literature on proteinuria and its association with immune‐mediated disease in dogs.

**Hypothesis:**

Renal proteinuria is common in dogs with immune‐mediated disease. Dogs presenting with pyrexia or immune‐mediated polyarthritis (IMPA) are more likely to have proteinuria.

**Animals:**

One hundred and forty‐four dogs with primary immune‐mediated diseases.

**Methods:**

Retrospective, observational study. Data collected included signalment, travel outside the United Kingdom, duration of clinical signs, diagnosis, urinalysis, and urine protein–creatinine ratio (UPCR). Non‐proteinuric, mild proteinuria, moderate proteinuria, and severe proteinuria were defined as UPCR < 0.5; ≥ 0.5–1; ≥ 1–2; ≥ 2, respectively. Exclusion criteria included azotemia, hypoalbuminemia (< 2.0 g/dL), foreign travel, active urine sediment or positive culture, glucocorticoid therapy for greater than 24 h prior to presentation, or medication known to influence UPCR.

**Results:**

Sixty‐seven dogs were non‐proteinuric (47%; 95% confidence interval [95% CI]: 38%, 55%), 25 mildly proteinuric (17%; 95% CI: 9%, 26%), 15 moderately proteinuric (10%; 95% CI: 2%, 19%), and 37 severely proteinuric (26%; 95% CI: 17%, 34%). On multiple logistic regression analysis, female dogs (odds ratio [OR]: 3.24; 95% CI: 1.49, 7.42), individuals with pyrexia (OR: 6.59; 95% CI: 3.00, 15.37), or hemoglobinuria (OR: 27.21; 95% CI: 4.79, 516.56) were more likely to have proteinuria. There was an association between steroid‐responsive meningitis‐arteritis and the magnitude of proteinuria on multiple linear regression (*p* = 0.025); this was not confirmed on multiple logistic regression.

**Conclusions and Clinical Importance:**

Proteinuria is common in dogs with immune‐mediated disease and can be severe. Screening for proteinuria could be considered part of the diagnostic assessment for dogs with immune‐mediated disease.

**Abbreviations**
ANA: antinuclear antibody
HPF: high‐power field
IMHA: immune‐mediated hemolytic anemia
IMN: immune‐mediated neutropenia
IMPA: immune‐mediated polyarthritis
IMTP: immune‐mediated thrombocytopenia
IRIS: International Renal Interest Society
SLE: systemic lupus erythematosus
SRMA: steroid‐responsive meningitis‐arteritis
UPCR: urine protein–creatinine ratio

## 1 Introduction

Proteinuria is defined as the presence of increased protein in the urine [1]. There are many possible causes, which can be categorized by the location or mechanism of the underlying abnormality. Cases are categorized on the site of disease into prerenal, renal, or postrenal. Prerenal proteinuria is due to abnormal plasma content of proteins that transverse normally permselective glomerular capillary walls. This can be due to proteins that are not normally present free in the plasma, such as hemoglobinuria in intravascular hemolysis, or the presence of abnormal proteins such as immunoglobulins light chains in malignant plasma cell neoplasms. Renal proteinuria can be further classified as functional or pathological. Functional proteinuria is defined as proteinuria that is due to altered renal physiology during or in response to transient phenomena such as strenuous exercise, pyrexia, and seizures and therefore not attributable to renal lesions. Conversely, pathological renal proteinuria is attributable to structural or functional lesions within the kidneys and can be further defined by the location of these lesions as either glomerular, tubular, or interstitial in origin. Finally, post‐renal proteinuria is the result of the entry of proteins derived from the urine excretory pathway, normally the result of an exudative or hemorrhagic process, for example, urinary tract infection, pyuria, hematuria [1].

In dogs and cats, persistent renal proteinuria is associated with several negative clinical outcomes, including increased risk of uremic crisis, renal disease progression, and death [2, 3, 4, 5, 6]. The persistence and magnitude of proteinuria often determine decisions regarding therapeutic intervention in dogs [7]. Nephropathies caused by immune‐mediated mechanisms are well documented in human medicine, the etiology of which can be broadly classified into two groups: whether they are caused by anti‐glomerular basement membrane antibodies or certain infectious or autoimmune stimuli resulting in deposition of circulating immunocomplexes or activation of complement or neutrophil extracellular traps within the glomeruli [8, 9, 10]. Proteinuria is associated with a wide range of human autoimmune diseases [11, 12, 13, 14, 15, 16, 17, 18]. Immune complex formation is a complication of canine immune‐mediated diseases such as immune‐mediated polyarthritis (IMPA) [19, 20, 21, 22]. Nephropathies and proteinuria occur in human patients with rheumatoid arthritis [16, 23] and the prevalence of proteinuria in dogs with IMPA is as high as 38% [24]. The International Renal Interest Society (IRIS) Canine Glomerulonephritis Study Group recommends that screening for immune‐mediated disease should be undertaken in dogs with proteinuria [25]. Despite this, information is limited on the association between proteinuria and immune‐mediated disease in dogs.

The objectives of this study were to estimate the prevalence and severity of proteinuria in dogs presenting to a referral hospital with immune‐mediated disease, and to evaluate the association between proteinuria and animal, disease, and therapy‐related variables. We hypothesized that renal proteinuria would be common in dogs with immune‐mediated disease and individuals presenting with pyrexia or IMPA would be more likely to have proteinuria.

## 2 Materials and Methods

### 2.1 Study Design and Ethical Approval

This was a retrospective observational study examining proteinuria in client‐owned dogs diagnosed with various immune‐mediated diseases. The University of Liverpool Committee on Research Ethics granted ethical approval prior to the study (VREC1116) and all owners gave their informed consent in writing.

### 2.2 Case Identification, Eligibility Criteria, and Data Collection

Medical records of all animals presenting to the University of Liverpool Small Animal Teaching Hospital between June 2011 and June 2021 were searched for specific immune‐mediated diseases including: immune‐mediated hemolytic anemia (IMHA), IMPA, immune‐mediated thrombocytopenia (IMTP), neutrophilic lymphadenitis, immune‐mediated neutropenia (IMN), polyradiculoneuritis, sterile panniculitis, steroid‐responsive meningitis‐arteritis (SRMA), pemphigus foliaceus, idiopathic hypertrophic pachymeningitis, granulomatous vasculitis, erythema multiforme, and meningoencephalitis of unknown origin (MUO). Details of each case, including the diagnosis, were reviewed by an EBVS European Veterinary Specialist in Internal Medicine.

Dogs were eligible if they were diagnosed with one of the previously listed immune‐mediated diseases. Dogs must have had urinalysis performed (urine sediment examination, dipstick, urine specific gravity [USG]) including urine protein–creatinine ratio (UPCR) during their initial investigations. Dogs were not eligible if they had azotemia, defined as a serum creatinine concentration > 1.4 mg/dL (as per the IRIS definition for chronic kidney disease [CKD]), a progressive non‐azotemic increase in serum creatinine concentration ≥ 0.3 mg/dL, documented within 48 h of hospitalization (as per the IRIS definition of non‐azotemic acute kidney injury [AKI]), or either measured oliguria or anuria (also as per the IRIS AKI definition) [26, 27]. Dogs were also excluded if they experienced severe hypoalbuminemia (< 2.0 g/dL) at presentation or at any time throughout hospitalization, had traveled outside of the United Kingdom or had either an active urine sediment, the presence of granular casts, or positive urine culture. An active urine sediment was defined as a sample in which ≥ 5 white blood cells (WBCs)/high‐power field (×40 objective), ≥ 10 red blood cells (RBCs) per high‐power field (×40 objective), or bacteria were observed, similar to previous studies [28, 29]. Further, dogs were not eligible if they had an underlying disease thought to trigger the immune‐mediated disease. Ineligible conditions included infectious diseases (e.g., endocarditis and vector‐borne diseases) or neoplasia, which can be associated with both proteinuria and immune‐mediated disease [25, 30, 31, 32, 33, 34, 35]. Finally, dogs that had received glucocorticoid therapy for > 24 h prior to presentation, or other medication known to influence UPCR, for example, tyrosine kinase inhibitors, were also excluded [36].

For all dogs, data collected included signalment, travel history, use of glucocorticoids prior to referral diagnosis, duration of clinical signs prior to presentation, urinalysis, and UPCR taken from a single urine sample.

### 2.3 Clinicopathological Investigations

All clinicopathological investigations were conducted by the University of Liverpool Clinical Pathology Service and Microbiology Diagnostics Laboratory. Peripheral blood was collected from a jugular vein of each dog, unless thrombocytopenia prevented safe acquisition, in which case a saphenous or cephalic vein was used as determined by clinician preference. Urine samples were acquired by ultrasound‐guided cystocentesis, unless precluded by severe thrombocytopenia (platelet count < 50 × 10^9^/L), in which case a free‐catch sample was acquired. Aerobic urine culture was performed in dogs with lower urinary tract signs and in those with an active sediment. Hemoglobinuria was considered to be present if causes of myoglobinuria were absent (and increased creatinine kinase or aspartate aminotransferase activity were not present on serum biochemistry), if there was a red discoloration of the urine that persisted after centrifugation, or if a positive heme reaction on urine dipstick was present in the absence of intact erythrocytes on microscopic sediment examination. Urine samples were refrigerated for no longer than 24 h before aerobic culture was performed. UPCR was performed using a biochemistry analyzer.^1^ Urine protein was measured using the pyrogallol red method and urine creatinine by the enzymatic method. Non‐proteinuric, mild proteinuria, moderate proteinuria, and severe proteinuria were defined as UPCR < 0.5; ≥ 0.5–1; ≥ 1–2; ≥ 2, respectively. Definitions of non‐proteinuria were based on the ACVIM consensus statement [1]. Severe proteinuria was defined as a UPCR ≥ 2 because such a magnitude is considered the result of glomerular disease. This is supported by the IRIS Canine Glomerulonephritis Study Group, which states that dogs exhibit more adverse outcomes if the UPCR exceeds 2.0 [1, 7]. Mild and moderate proteinuria definitions were arbitrary.

### 2.4 Data Handling and Statistical Analysis

Initially, data was entered into an electronic spreadsheet (Microsoft Excel) and checked for errors. An online, open‐access statistical language and environment (R, version 4.3.1) [37], was then used for all statistical analyses, with several additional packages including aod (version 1.3.2) [38], car (version 3.1.2) [39], DescTools (version 0.99.58) [40], glmtoolbox (version 0.1.7) [41], MASS (version 7.3.60) [42], and performance (version 0.10.4) [43]. The prevalence of proteinuria was calculated by dividing the portion of mild to severely proteinuric dogs by the total number of dogs, then multiplying by 100 to report as a percentage. Descriptive statistics for quantitative variables (age, bodyweight, duration of clinical signs) were expressed as median (range), while categorical variables were expressed as number and percentage. Further, differences in the magnitude of proteinuria between dogs with IMHA with and without hemoglobinuria were assessed with a Mann–Whitney test.

Simple and multiple linear regression were used to determine variables associated with the magnitude of proteinuria, with the lm function in R. For these analyses, UPCR was first logarithmically transformed because this improved model fit and also ensured that the assumptions of the model were met (see below). Explanatory variables tested included age, sex, neuter status, breed, duration of clinical signs before diagnosis, presence of pyrexia, presence of hemoglobinuria, and prior use of glucocorticoids. Age and duration of clinical signs were included as continuous variables, while the remaining explanatory variables were included as nominal categorical variables. Continuous predictor variables were logarithmically transformed if this improved model fit and performance. Most categorical variables were included in a binary form (e.g., sex, neuter status, pyrexia, hemoglobinuria, and use of glucocorticoids); for sex and neuter status, the referent categories were male and intact, respectively; all other variables were classified as yes–no, and no was chosen as the referent category. For breed, given many breeds represented, breed groups were first created containing similar breeds; these included: Cocker spaniel (including English and American varieties), Springer spaniel (including English and Welsh varieties), retriever (including Golden Retriever, Labrador Retriever, Flat‐Coated Retriever), sight hound (including Greyhound, Lurcher, Whippet), Mixed breed and other (including Bichon frise, Border collie, Boxer, English bulldog, Fox terrier, German shepherd dog, Great Dane, Irish setter, Jack Russell terrier, Maltese terrier, Miniature Dachshund, Miniature Schnauzer, Pomeranian, Pug, Rottweiler, Shih tzu, Siberian husky, Staffordshire bull terrier, Toy poodle, Weimaraner, West Highland white terrier, Yorkshire terrier). A nominal categorical variable was then created where mixed breed was the referent category. For immune‐mediated disease, given that some individuals had more than one disease diagnosed, separate binary variables (present vs. absent) were used for the different immune‐mediated diseases; for each, absent was chosen as the referent category. The only between‐variable interaction tested was between sex and neuter status; no other interactions were tested because none were deemed to be clinically relevant.

Initially, a series of simple models were created with log(UPCR) as the outcome variable and single explanatory variables (except for disease, as explained above). A multiple regression model was built that initially included all variables that were *p* < 0.200 on simple regression. This model was then refined in a backward and forward stepwise fashion, with the Bayesian information criterion (BIC; a measure of its goodness of fit compared with its complexity) being used to select the model within the same family with the best generalizability [44]. With this approach, the existing model was repeatedly refined with the addition or removal of variables until the model with the smallest BIC was found, according to previously published rules [45]. Model assumptions were tested in various ways. Normality of residuals was tested by visually inspecting histograms and Q–Q plots and using the Shapiro–Wilk test. Homogeneity of variance was tested using visual inspection (of a plot of fitted values against the square root of the standardized residuals) and the Breusch–Pagan test; autocorrelation was tested using the Durbin–Watson test. Influential data points were identified and assessed using Cook's distance; since no influential points were identified, it was not necessary to consider removing any data points. Possible multicollinearity, in models containing multiple explanatory variables, was assessed using variance inflation factors (VIFs) and was considered acceptable when all values were < 4. If necessary, multicollinearity was resolved by removing the variable with the greatest VIF and repeating the analysis. Model performance was assessed using adjusted *R* ^2^ and the associated *p* value.

Simple and multiple binary logistic regression models were also created, using the “glm” function in R, to determine associations between predictor variables and the presence of proteinuria. The outcome variable was presence of proteinuria (defined as UPCR > 0.5), while the same predictor variables were used as described for linear regression. Once again, the only interaction tested was between sex and neuter status and, again, continuous predictor variables were first logarithmically transformed if this improved model fit and performance. Simple and multiple logistic models were created and refined using BIC, as described for linear regression. Influential datapoints and possible multicollinearity were assessed and resolved in the same way as for linear regression. To assess model fit, the deviance of the model was first compared with the null deviance using a chi square test; for explanatory variables with more than two levels, goodness of fit was also assessed by visual inspection of observed and expected results and the Hosmer–Lemeshow test. Model performance was assessed by calculating the coefficient of determination (pseudo *R* ^2^) of the overall model, using the method of Nagelkerke [46]. Results from both simple and multiple regression are reported as odds ratios (OR), 95% confidence intervals (95% CI) and the associated *p* value (based upon the Wald chi‐square test).

## 3 Results

### 3.1 Characteristics of Study Dogs

Full details of the 144 dogs that met the enrollment criteria are given in Table 1. The number of male and female dogs was broadly similar, and most were neutered. There was a wide age range, from 6 months to 12 years, and a range of breeds was represented, with the most common being mixed breed, English springer spaniel, Cocker spaniel, Whippet, and Labrador retriever. The median duration of clinical signs prior to presentation was ten days (range 1–168 days). Sixty‐one dogs (42%) were pyrexic on presentation, 16 dogs (11%) had hemoglobinuria, 15 of which had IMHA, and the remaining dog had polyradiculoneuritis. Eleven dogs (8%) had received glucocorticoid therapy within 24 h of presentation at the referral hospital.

**TABLE 1:** Signalment of the 144 study dogs with confirmed immune‐mediated disease.

Variable	Results
Age (months)	49 (12–152)
Sex	
Male intact	19 (13%)
Male neutered	56 (39%)
Female intact	14 (9%)
Female neutered	55 (38%)
Breed	Airedale (1), American cocker spaniel (1), Bichon Frise (2), Border collie (6), Border terrier (1), Boxer (1), Cocker spaniel (13), English Bulldog (1), English springer spaniel (16), Flat‐coated retriever (1), Fox terrier (1), German shepherd dog (3), Golden retriever (1), Great Dane (1), Greyhound (5), Irish setter (2), Jack Russell terrier (6), Labrador retriever (8), Lurcher (4), Maltese terrier (1), Miniature dachshund (1), Miniature schnauzer (7), mixed breed (30), Pomeranian (1), Pug (1), Rottweiler (1), Shih Tzu (7), Siberian Husky (1), Staffordshire Bull Terrier (2), Toy poodle (1), Weimaraner (1), Welsh springer spaniel (1), Welsh terrier (1), West Highland white terrier (3), Whippet (9), Yorkshire terrier (2)
Immune‐mediated disease^a^	
IMHA	37 (26%)
IMTP	10 (7%)
IMPA	44 (31%)
SRMA	21 (15%)
Neutrophilic lymphadenitis	13 (9%)
Other	22 (15%) Erythema multiforme (1), granulomatous vasculitis (1), idiopathic hypertrophic pachymeningitis (1), Immune‐mediated neutropenia (5), pemphigus foliaceus (3), polyradiculoneuritis (7), sterile panniculitis (4)
Pyrexia present	61 (42%)
Hemoglobinuria	16 (11%)
Duration of signs (days)	10 (1 to 168)
Glucocorticoids administered^b^	14 (10%)

Abbreviations: IMHA, immune‐mediated hemolytic anemia; IMN, immune‐mediated neutropenia; IMPA, immune‐mediated polyarthritis; IMTP, immune‐mediated thrombocytopenia; SRMA, steroid responsive meningitis arteritis.

^a^ Please note that the total number of diseases is 147, because one dog had concurrent IMHA and IMTP, while two dogs had concurrent IMPA and SRMA; all other dogs had a single immune‐mediated disease.

^b^ Glucocorticoids administered within 24 h of diagnosis.

### 3.2 Immune‐Mediated Diseases

Thirteen different immune‐mediated diseases were diagnosed, with IMPA, IMHA, and SRMA being the most common. The diagnoses and their respective prevalence and severities of proteinuria are summarized in Table 2.

**TABLE 2:** Severity and prevalence of proteinuria respective to specific immune‐mediated diseases in 144 dogs.

Disease	Median UPCR	No. dogs^a^	No. (%) with proteinuria (UPCR > 0.5)	Non‐proteinuric^b^ (UPCR < 0.5)	Mild proteinuria^b^ (UPCR ≥ 0.5–1)	Moderate proteinuria^b^ (UPCR ≥ 1–2)	Severe proteinuria^b^ (UPCR ≥ 2)
IMHA							
No hemoglobinuria^c^	0.49 (0.08–14.10)	22	10 (45%)	12 (55%)	2 (9%)	0 (0%)	8 (36%)
With hemoglobinuria^c^	4.24 (0.18–20.30)	15	14 (93%)	1 (7%)	0 (0%)	3 (20%)	11 (73%)
IMTP	0.22 (0.08–0.93)	10	2 (20%)	8 (80%)	2 (20%)	0 (0%)	0 (0%)
SRMA	0.38 (0.02–3.30)	21	9 (45%)	12 (55%)	6 (30%)	2 (10%)	1 (5%)
IMPA	0.69 (0.08–9.73)	44	24 (56%)	20 (44%)	9 (21%)	6 (14%)	9 (21%)
Neutrophilic lymphadenitis	0.58 (0.08–3.13)	13	7 (54%)	6 (46%)	2 (15%)	3 (23%)	2 (15%)
IMN	0.78 (0.05–1.63)	5	3 (60%)	2 (40%)	2 (40%)	1 (20%)	0 (0%)
Granulomatous vasculitis	4.74	1	1 (100%)	0 (0%)	0 (0%)	0 (0%)	1 (100%)
Erythema multiforme	4.47	1	1 (100%)	0 (0%)	0 (0%)	0 (0%)	1 (100%)
Polyradiculoneuritis	0.25 (0.14–0.95)	7	2 (29%)	5 (71%)	2 (29%)	0 (0%)	0 (0%)
Sterile panniculitis	1.62 (0.08–3.10)	4	3 (75%)	1 (25%)	1 (25%)	0	2 (50%)
Pemphigus foliaceus	0.12 (0.06–11.80)	3	1 (33%)	2 (67%)	0 (0%)	0 (0%)	1 (33%)
Idiopathic hypertrophic pachymeningitis	4.35	1	1 (100%)	0 (0%)	0 (0%)	0 (0%)	1 (100%)

Abbreviations: IMHA, immune‐mediated hemolytic anemia; IMN, immune‐mediated neutropenia; IMPA, immune‐mediated polyarthritis; IMTP, immune‐mediated thrombocytopenia; SRMA, steroid responsive meningitis arteritis.

^a^ Please note that the total number of diseases is 147, because one dog had concurrent IMHA and IMTP, while two dogs had concurrent IMPA and SRMA; all other dogs had a single immune‐mediated disease.

^b^ Non‐proteinuric = UPCR < 0.5; mild proteinuria = UPCR ≥ 0.5–1; moderate proteinuria = UPCR ≥ 1–2; severe proteinuria = UPCR ≥ 2; prevalence calculated by dividing the portion of mild‐to‐severely proteinuric patients by the total patient number within the individual breed, and then multiplying by 100 to create a percentage.

^c^ Hemoglobinuria is defined by having a positive response on dipstick to blood and an inactive sediment without evidence of erythrocytes on urine microscopic examination.

### 3.3 Magnitude and Prevalence of Proteinuria

Most (77 dogs, 53%; 95% CI: 45%, 62%) dogs had proteinuria, with the median UPCR being 0.63 (range 0.02–20.30). Twenty‐five dogs (17%; 95% CI: 9%, 26%) were mildly proteinuric (UPCR ≥ 0.5–1.0), 15 dogs (10%; 95% CI: 2%, 19%) were moderately proteinuric (UPCR ≥ 1.0–2.0) and 37 dogs (26%; 95% CI: 17%, 34%) were severely proteinuric (UPCR ≥ 2.0). The magnitude of proteinuria in dogs with IMHA and concurrent hemoglobinuria (4.24, 0.18–20.30) was greater than that in IMHA dogs without concurrent hemoglobinuria (0.49. 0.08–14.10, *p* = 0.002; Table 2).

### 3.4 Linear Regression Analysis to Determine Variables Associated With Magnitude of Proteinuria

Results of simple and multiple linear regression analysis, to determine variables associated with the magnitude of proteinuria, are shown in Tables 3 and 4, respectively. In preliminary analyses, a decision was made to logarithmically transform UPCR results, because this improved model fit and ensured that model assumptions were met. Further, model fit for duration of clinical signs was improved when this predictor variable was logarithmically transformed. On simple linear regression analysis (Table 3), the following variables met the threshold (*p* < 0.200) for initial inclusion in best‐fit multiple regression modeling: sex (adjusted *R* ^2^: 0.046, *p* = 0.006), IMHA (adjusted *R* ^2^: 0.090, *p* < 0.001), IMTP (adjusted *R* ^2^: 0.029, *p* = 0.024), SRMA (adjusted *R* ^2^: 0.024, *p* = 0.034), log(duration of clinical signs; adjusted *R* ^2^: 0.049, *p* < 0.001), pyrexia present (adjusted *R* ^2^: 0.068, *p < 0*.001), hemoglobinuria (adjusted *R* ^2^: 0.169, *p* < 0.001), and administration of glucocorticoids in the 24 h prior to referral (adjusted *R* ^2^: 0.016, *p* = 0.069). After refinement by backwards and forwards stepwise elimination, the model with the best generalizability (adjusted *R* ^2^: 0.332, *p* < 0.001; Table 4) included these predictor variables: sex (*p* = 0.040), pyrexia present (*p* < 0.001), hemoglobinuria (*p* < 0.001) and SRMA (*p* = 0.025). Being a female dog (regression coefficient 0.424, 95% CI: 0.02, 0.83), having pyrexia (regression coefficient 1.13, 95% CI: 0.72, 1.54) and having hemoglobinuria (regression coefficient 2.03, 95% CI: 1.38, 2.67) were all positively associated with the magnitude of pyrexia, while having SDMA was negatively associated (regression coefficient−0.67, 95% CI: −1.25, −0.09).

**TABLE 3:** Simple linear regression analysis to determine variables associated with the magnitude of proteinuria^a^ in 144 dogs with immune‐mediated disease.

Variable	Estimate^b^	95% Confidence interval (CI)^b^	Adjusted *R* ^2^ ^c^	BIC^d^	*p*
Age (months)^e^	0.004	−0.003, 0.011	0.003	408	0.235
Sex	—	—	0.046	401	**0.006**
Male (ref)	—	—	—		—
Female	0.667	0.198, 1.137	—	—	—
Neuter status					
Intact (ref)	—	—	—	—	—
Neutered	−0.051	−0.625, 0.523	−0.007	409	0.861
Breed	—		−0.009	412	0.600
Mixed breed (ref)	—	—	—	—	—
Cocker spaniel	0.506	−0.432, 1.444	—	—	—
Retriever group	−0.389	−1.448, 0.669	—	—	—
Sight hound group	0.199	−0.665, 1.063	—	—	—
Springer spaniel group	−0.336	−1.216, 0.543	—	—	—
Other	−0.053	−0.711, 0.604	—	—	—
Immune‐mediated disease^f^ (present vs. absent [reference])					
IMHA	1.033	0.509, 1.558	0.090	395	**< 0.001**
IMPA	−0.016	−0.539, 0.508	−0.007	409	0.953
IMTP	−1.075	−2.007, −0.144	0.029	404	**0.024**
Neutrophilic lymphadenitis	−0.315	−1.155, 0.525	−0.003	409	0.459
SRMA	−0.728	−1.401, −0.056	0.024	405	**0.034**
Other	−0.168	−0.834, 0.502	−0.005	409	0.621
Log(duration of signs) (days)^e^	−0.291	−0.490, −0.092	0.049	401	**0.005**
Pyrexia^g^	0.803	0.331, 1.272	0.068	398	**< 0.001**
Hemoglobinuria^g^	1.935	1.238, 2.632	0.169	381	**< 0.001**
Glucocorticoids administered^h^	0.747	−0.058, 1.551	0.016	406	**0.069**

*Note:* *p* values in bold are those that met the threshold for inclusion in the multiple linear regression modeling (Table 4).

^a^ Urine protein–creatinine ratio results were logarithmically transformed because this improved model fit and also ensured that model assumptions were met.

^b^ Estimate and 95% CI of the regression coefficient for the predictor variable; given that the outcome variable was logarithmically transformed, the coefficients represent the expected difference in log(UPCR) for most predictor variable comparisons; taking sex as an example, the difference in mean UPCR between male and female dogs is exp(0.667) = 1.948; the exceptions are age and duration of signs because these were also logarithmically transformed, meaning that the coefficient represents the expected change in UPCR for each unit increase in the predictor; for example, a 1‐month age increase is associated with an increase in UPCR of 0.004.

^c^ Model performance assessed by calculating *R* ^2^ adjusted for the number of predictors in the model.

^d^ Model generalizability determined using Bayesian information criterion (BIC), with models having the best fit having lower BIC [33]. Please note that BIC values can only be compared within the same family of models.

^e^ Age and duration of clinical signs were logarithmically transformed because this improved model fit.

^f^ Immune‐mediated diseases diagnosed including immune‐mediated hemolytic anemia (IMHA), immune‐mediated polyarthritis (IMPA), immune‐mediated thrombocytopenia (IMTP), steroid‐responsive meningitis arteritis (SRMA), and other; see Table 1 for details of the immune‐mediated diseases included in the “other” group; please note that separate binary variables were used for each condition because some dogs were diagnosed with more than one disease; therefore, the referent category for each is absence of the disease.

^g^ Pyrexia and hemoglobinuria tested as binary variables (yes vs. no) with no being the referent category.

^h^ Glucocorticoids administered within 24 h of diagnosis tested as a binary variable (yes vs. no), with no again being the referent category.

**TABLE 4:** Best‐fit multiple linear regression model assessing variables associated with the magnitude of proteinuria^a^ in 144 dogs with immune‐mediated disease.

Variable	Estimate^b^	95% Confidence interval (CI)^b^	Adjusted *R* ^2^ ^c^	Bayesian information criterion (BIC)^d^	*p*
Final model details	—	—	0.332	362	**< 0.001**
*Predictor variables*					
Sex					
Male (reference)	—	—	—	—	—
Female	0.424	0.020, 0.829	—	—	**0.040**
SRMA diagnosed	−0.667	−1.249, −0.085	—	—	**0.025**
Pyrexia present	1.132	0.722, 1.543	—	—	**< 0.001**
Hemoglobinuria	2.027	1.383, 2.670	—	—	**< 0.001**

*Note:* *p* values in bold are those that met the threshold for significance of *p* < 0.05.

^a^ Urine protein–creatinine ratio results were logarithmically transformed because this improved model fit and also ensured that model assumptions were met.

^b^ Estimate and 95% CI of the regression coefficient for the predictor variable; given that the outcome variable was logarithmically transformed, the coefficients represent the expected difference in log(UPCR) for most predictor variable comparisons; taking sex as an example, the difference in mean UPCR between male and female dogs is exp(0.424) = 1.528.

^c^ Model performance was assessed by calculating the *R* ^2^ adjusted for the number of predictors in the model.

^d^ Model generalizability determined using BIC, with models having the best fit having lower BIC [33]. Please note that BIC values can only be compared within the same family of models.

### 3.5 Binary Logistic Regression Analysis to Determine Variables Associated With Presence of Proteinuria

Results of simple and multiple logistic regression analysis, to determine variables associated with the presence of proteinuria (UPCR > 0.5), are shown in Tables 5 and 6, respectively. In preliminary analyses, model fit for duration of clinical signs was again improved when these predictor variables were logarithmically transformed. With simple logistic regression analysis (Table 5), the following variables met the threshold (*p* < 0.200) for initial inclusion in best‐fit multiple regression modeling: sex (pseudo *R* ^2^: 0.084, *p* = 0.003), breed (pseudo *R* ^2^: 0.053, *p* = 0.12), IMHA (pseudo *R* ^2^: 0.024, *p = 0*.110), IMTP (pseudo *R* ^2^: 0.046, *p* = 0.045), log(duration of clinical signs; pseudo *R* ^2^: 0.036, *p* = 0.052), pyrexia present (pseudo *R* ^2^: 0.134, *p* < 0.001), and hemoglobinuria (pseudo *R* ^2^: 0.125, *p* = 0.008). After refinement by backward and forward stepwise elimination, the model with the best generalizability (adjusted *R* ^2^: 0.358, *p* = 0.004; Table 6) included these predictor variables: sex (*p* = 0.004), pyrexia present (*p* < 0.001) and hemoglobinuria (*p* = 0.002). Being a female dog (OR: 3.24; 95% CI: 1.49, 7.42), having pyrexia (OR: 6.59; 95% CI: 3.00, 15.37) and having hemoglobinuria (OR: 27.21; 95% CI: 4.79, 517.56) were all positively associated with the presence of pyrexia.

**TABLE 5:** Simple logistic regression to determine variables associated with the presence of proteinuria (UPCR > 0.5) in 144 dogs with immune‐mediated disease.

Variable	Odds ratio	95% Confidence interval (CI)^a^	Pseudo *R* ^2^ ^b^	Bayesian information criterion (BIC)^c^	*p*
Age (months)	1.000	0.991, 1.010	0.000	209	0.863
Sex					
Male (reference)	—	—	—	—	—
Female	2.839	1.451, 5.669	0.084	199	**0.003**
Neuter status					
Intact (reference)	—	—	—	—	—
Neutered	0.806	0.363, 1.759	0.003	209	0.591
Breed	—	—	0.053	223	**0.12**
Mixed breed (ref)	—	—	—	—	—
Cocker spaniel	3.208	0.807, 16.355	—	—	0.119
Retriever group	0.583	0.126, 2.464	—	—	0.468
Sight hound group	1.375	0.422, 4.657	—	—	0.599
Springer spaniel group	0.984	0.297, 3.292	—	—	0.979
Other	0.784	0.319, 1.913	—	—	0.594
Immune‐mediated disease^d^ (present vs. absent [reference])					
Immune‐mediated hemolytic anemia (IMHA)	1.881	0.879, 4.171	0.024	206	**0.110**
Immune‐mediated polyarthritis (IMPA)	1.064	0.523, 2.181	0.000	209	0.864
Immune‐mediated thrombocytopenia (IMTP)	0.197	0.029, 0.820	0.046	204	**0.045**
Neutrophilic lymphadenitis	1.017	0.321, 3.313	0.000	209	0.977
Steroid‐responsive meningitis arteritis (SRMA)	0.607	0.232, 1.537	0.010	208	0.294
Other	1.052	0.423, 2.668	0.000	209	0.913
Log(duration of signs) (days)	0.991	0.977, 1.003	0.036	205	**0.156**
Pyrexia^e^	3.922	1.953, 8.147	0.134	194	**< 0.001**
Hemoglobinuria^e^	15.968	3.096, 293.010	0.125	195	**0.008**
Glucocorticoids administered^f^	1.641	0.537, 5.587	0.07	208	0.397

*Note:* *p* values in bold are those that met the threshold for inclusion in the multiple logistic regression modeling (Table 4).

^a^ 95% CIs for the odds ratio.

^b^ Model performance was assessed by calculating the coefficient of determination (pseudo *R* ^2^) of the overall model, using the method of Nagelkerke [35].

^c^ Model generalizability determined using BIC, with models having the best fit having lower BIC [33]. Please note that BIC values can only be compared within the same family of models.

^d^ Immune‐mediated diseases diagnosed in the dogs including IMHA, IMPA, IMTP, SRMA, and others; see Table 1 for details of the immune‐mediated diseases included in the “other” group; please note that separate binary variables were used for each condition because some dogs were diagnosed with more than one disease; therefore, the referent category for each is absence of the disease.

^e^ Pyrexia and hemoglobinuria tested as binary variables (yes vs. no) with no being the referent category.

^f^ Glucocorticoids administered within 24 h of diagnosis, tested as a binary variable (yes vs. no), with “no” again being the referent category.

**TABLE 6:** Best‐fit multiple logistic regression model assessing variables associated with the presence of proteinuria (UPCR > 0.5) in 144 dogs with immune‐mediated disease.

Variable	Odds ratio	95% Confidence interval (CI)^a^	Pseudo *R* ^2^ ^b^	Bayesian information criterion (BIC)^c^	*p*
Final model details	—	—	0.358	174	**0.004**
*Predictor variables*					
Sex					
Male (reference)	—	—	—	—	—
Female	3.243	1.487, 7.416	—	—	**0.004**
Pyrexia present	6.590	3.005, 15.365	—	—	**< 0.001**
Hemoglobinuria	27.206	4.791, 517.558	—	—	**0.002**

*Note:* *p* values in bold are those that met the threshold for significance of *p* < 0.05.

^a^ 95% CIs for the odds ratio.

^b^ Model performance was assessed by calculating the coefficient of determination (pseudo *R* ^2^) of the overall model, using the method of Nagelkerke [35].

^c^ Model generalizability determined using BIC, with models having the best fit having lower BIC [33]. Please note that BIC values can only be compared within the same family of models.

## 4 Discussion

In humans, proteinuria is associated with several autoimmune diseases including systemic lupus erythematosus (SLE), rheumatoid arthritis, and autoimmune thyroiditis (Grave's disease) [11, 12, 13, 14, 15, 16, 17, 18]. In the current study, proteinuria was common in dogs with immune‐mediated diseases, a quarter of which had severe proteinuria. Further, the odds of having proteinuria were greater in female dogs, those with pyrexia or hemoglobinuria, and those with SRMA. There is limited research about the presence of proteinuria in dogs with immune‐mediated disease, making comparisons with the current study difficult. However, the point prevalence of proteinuria in one study of dogs with IMPA [24], was less than in the current study (38% vs. 55%). The reason for the discrepancy is unclear, with possibilities including differences in the duration of clinical signs and prior use of glucocorticoid therapy; in the previous study [24], dogs were excluded if they had any history of glucocorticoid administration, while those with < 24 h administration were eligible in the current study. Alternatively, the difference in prevalence might reflect the greater range of conditions included in the current study; indeed, the prevalence of severe proteinuria (UPCR ≥ 2) in the dogs with IMPA in our study (21%) was similar to that seen in the previous study (20%) [24]. Nevertheless, since both studies were undertaken in different locations, were retrospective in nature, and eligibility criteria differed, further comparisons between the two cohorts should be made cautiously [24].

The pathophysiology of proteinuria in dogs with immune‐mediated disease has not been fully characterized and remains incompletely understood. SLE, rheumatoid arthritis, and IMPA are examples of type III hypersensitivity reactions in people and dogs, respectively. Therefore, the pathophysiology of proteinuria in these cases might be associated with immune complex deposition in the glomerular basement membrane. Alternatively, the proteinuria associated with immune‐mediated disease might be secondary to inflammation, not least since some immune‐mediated diseases in dogs (e.g., IMHA, SRMA and IMPA) are characterized by cytokine dysregulation, development of pyrexia, and activation of acute phase proteins [47, 48, 49, 50, 51, 52]. Further, IMHA in dogs has a similar cytokine profile to sepsis [52], while SRMA is associated with a systemic inflammatory response and the overproduction of cytokines interleukin (IL)‐6, IL‐17, transforming growth factor (TGF)‐β, vascular endothelial growth factor (VEGF) and CC‐motif ligand 19 (CCL19) [53]. Cytokines cause glomerular dysfunction in human medicine, and this might also be the case for dogs [54]. Cytokines, being low molecular weight proteins, are freely filtered at the glomerulus [55], while production of cytokines in severe inflammatory states could also saturate the resorptive channels in the proximal convoluted tubules leading to overflow proteinuria. For this reason, we hypothesized that pyrexia would be positively correlated with duration and severity of a pro‐inflammatory state and subsequent cytokine production, glomerular dysfunction, and overflow proteinuria. This was confirmed using both linear and logistic regression analysis; pyrexia was a risk factor in the final multiple regression models using both techniques. Further, while not significant in multiple logistic regression, SRMA was also a risk factor for proteinuria in multiple linear regression, adding further support to this theory. The use of electrophoresis or mass spectrometry was not feasible in this study but could be used to further evaluate the origin of the proteinuria in dogs with immune‐mediated disease in future research. A comparison of cytokine profiles in such might also be of interest.

The presence of hemoglobinuria, as a risk factor for proteinuria, was not unexpected because hemoglobin will transverse glomerular capillary walls even when they have normal permselective properties consistent with pre‐renal proteinuria. However, although the magnitude of proteinuria was greater in IMHA dogs with hemoglobinuria, compared with those without hemoglobinuria, almost half (45%) of those in the latter group still had proteinuria, as defined by a UPCR > 0.5. This finding suggests that hemoglobinuria is not the only reason for proteinuria in dogs with IMHA, with other possible reasons being systemic inflammation and cytokine dysregulation, as discussed above. Besides dogs with IMHA, the only other case that presented with apparent hemoglobinuria was a dog with polyradiculoneuritis, but the reason for this is unclear. One possibility could be the fact that the presence of RBCs was missed on sediment analysis.

In our cohort, female dogs were more likely to have proteinuria than male dogs. There is a marked female predisposition for autoimmune diseases in human medicine, where approximately 80% of patients diagnosed are women [56]. Although the reason for this remains unknown, it is postulated to be multifactorial, including an association with sex hormone changes in women and the association of autoimmune conditions with the X chromosome and X inactivation [57]. Although female dogs are overrepresented in studies on IMHA and MUO [58, 59, 60], a clear female predisposition in all immune‐mediated diseases has not definitively been proven in dogs. To the authors knowledge, no sex predisposition has previously been identified in canine glomerular disease; however, in human medicine, males are predisposed, except for lupus nephritis, where there is a marked female predisposition [61]. Given that testing for antinuclear antibodies (ANAs) was not performed in the current study, it is possible that some dogs might have had SLE and lupus nephritis. However, ANA testing has substantial limitations in veterinary medicine [62]. Furthermore, dogs that are biopsied for evaluation of renal proteinuria rarely have either conditions [63]. An alternative explanation for the female sex predisposition seen in the current study might be that disease severity is greater in female dogs.

Screening for and treatment of proteinuria in dogs is important. Proteinuria is a poor prognostic indicator in dogs and cats with CKD. In azotemic dogs, a UPCR > 1 is associated with increased risk of uremic crisis and death [4, 5]. A high UPCR is a negative prognostic factor in dogs with glomerular diseases and leishmaniasis [6]. Response to treatment with angiotensin‐converting enzyme inhibitors in dogs with a UPCR > 2.0 conveys a significant survival benefit [64]. The role proteinuria has on prognosis in dogs with immune‐mediated disease was beyond the scope of this study. Dogs with idiopathic non‐erosive IMPA and marked proteinuria (UPCR > 2.0) can show significant improvement in their UPCR with prednisolone in spite of the known contribution of the drug to proteinuria in dogs [24]. However, as in our study, the effect of proteinuria on survival has not been assessed.

This study had several limitations beyond its retrospective design. First, this was a referral caseload and might not reflect the caseload of dogs with immune‐mediated disease presenting to primary care practices. Second, there was a possible limitation regarding the definition of proteinuria; given possible day‐to‐day variation, the American College of Veterinary Internal Medicine (ACVIM) consensus group study recommends confirming consistent renal proteinuria by obtaining three samples for measurement 2 or more weeks apart [1]. However, this approach was not followed in the current study and, as a result, we cannot rule out the possibility of false positives and negatives. With that being said, the aim of this study was to assess for the presence of proteinuria, not its persistence. Third, based on the selection criteria, dogs with azotemia consistent with IRIS definitions of AKI and CKD were excluded. However, this does not necessarily exclude all cases of non‐azotemic renal disease, including dogs with a previous history of AKI or documented morphological changes on renal imaging. Therefore, while every effort was made to exclude such cases, some might have been missed. Fourth, many cases received immunosuppressive therapy with glucocorticoids, which can be associated with significant proteinuria in dogs [65, 66]. Interestingly, despite receiving glucocorticoids, the magnitude of proteinuria decreased in 9 dogs diagnosed with idiopathic non‐erosive IMPA where UPCR was originally > 2.0 [24]. Fifth, we did not perform either indirect blood pressure measurement or renal biopsy in any of the study dogs. As a result, the exact cause and location of the proteinuria could not be ascertained, nor can we entirely exclude the possibility of unidentified hypertension contributing to the observed proteinuria. Sixth, the cohort comprised a heterogeneous group of dogs with immune‐mediated diseases, which are likely to have different biological behaviors and prognoses. Unfortunately, comparison between diseases was limited by case number, not least for some diseases such as hypertrophic pachymeningitis and IMN.

In conclusion, proteinuria is common in dogs with immune‐mediated disease. Overall, the magnitude of proteinuria is mild, although severe proteinuria was sometimes seen, even in the absence of pigmenturia. The odds of having proteinuria were positively associated with being female, having pyrexia, and having SRMA. Although both the cause and significance of the observed proteinuria are not known, our results support screening for proteinuria as part of the diagnostic assessment of dogs presenting with immune‐mediated disease. They also provide a baseline for future prospective studies to assess the course of proteinuria in response to glucocorticoid therapy, whether medical treatment of proteinuria is required at initial diagnosis, and the role of proteinuria as a prognostic marker in dogs with immune‐mediated disease.

## Disclosure

Authors declare no off‐label use of antimicrobials.

## Ethics Statement

Approved by the University of Liverpool Veterinary Research Ethics Committee, Reference VREC1116. Authors declare human ethics approval was not needed.

## Conflicts of Interest

Dr. Alexander J.German is an employee of the University of Liverpool, but his post is financially supported by Royal Canin. Dr. Alexander J. German has also received financial remuneration for providing educational material, speaking at conferences, and consultancy work from this company; all such remuneration has been for projects unrelated to the work reported in this manuscript. The other authors declare no conflicts of interest.

## Endnotes

^1^
AU480 Chemistry Analyzer, Beckman Coulter UK Ltd., High Wycombe, Buckinghamshire, UK.
